# Mildew Locus O facilitates colonization by arbuscular mycorrhizal fungi in angiosperms

**DOI:** 10.1111/nph.16465

**Published:** 2020-02-28

**Authors:** Catherine N. Jacott, Myriam Charpentier, Jeremy D. Murray, Christopher J. Ridout

**Affiliations:** ^1^ Crop Genetics Department John Innes Centre Norwich Research Park Norwich NR4 7UH UK; ^2^ Cell and Developmental Biology Department John Innes Centre Norwich Research Park Norwich NR4 7UH UK; ^3^ National Key Laboratory of Plant Molecular Genetics CAS‐JIC Centre of Excellence for Plant and Microbial Science (CEPAMS) CAS Centre for Excellence in Molecular and Plant Sciences Institute of Plant Physiology and Ecology Chinese Academy of Sciences Shanghai 200032 China

**Keywords:** arbuscular mycorrhizal fungi, barley, *Medicago truncatula*, Mildew Resistance Locus O (MLO), powdery mildew

## Abstract

- Loss of barley *Mildew Resistance Locus O* (*MLO*) is known to confer durable and robust resistance to powdery mildew (*Blumeria graminis*), a biotrophic fungal leaf pathogen. Based on the increased expression of *MLO* in mycorrhizal roots and its presence in a clade of the MLO family that is specific to mycorrhizal‐host species, we investigated the potential role of MLO in arbuscular mycorrhizal interactions.
- Using mutants from barley (*Hordeum vulgare*), wheat (*Triticum aestivum*), and *Medicago truncatula*, we demonstrate a role for MLO in colonization by the arbuscular mycorrhizal fungus *Rhizophagus irregularis*.
- Early mycorrhizal colonization was reduced in *mlo* mutants of barley, wheat, and *M. truncatula*, and this was accompanied by a pronounced decrease in the expression of many of the key genes required for intracellular accommodation of arbuscular mycorrhizal fungi.
- These findings show that clade IV MLOs are involved in the establishment of symbiotic associations with beneficial fungi, a role that has been appropriated by powdery mildew.

## Abstract 2

See also the Commentary on this article by Zuccaro & Langen, **227**: 279–282.

## Introduction

Plants have co‐evolved with microbes, which is evident in the elaborate strategies that plants use to both promote beneficial symbioses and to restrict pathogenesis. For example, to establish beneficial endosymbioses like arbuscular mycorrhization, plants have dedicated host signalling pathways involving hundreds of genes (Oldroyd, 2013; Bravo *et al.*, 2016). Similarly, to keep apace with pathogens, components of the plant immune system are rapidly evolving and expanding (Chisholm *et al.*, 2006; Han, 2019). Pathogens employ strategies to take advantage of host genes – so‐called ‘susceptibility factors’ – to cause disease (O'Connell & Panstruga, 2006; Boevink *et al.*, 2016). One example of a susceptibility factor is the barley *Mildew resistance locus O* (*MLO)* gene – referred to here as *MLO1* to distinguish it from other family members – which is required for successful colonization by powdery mildew fungi, *Erysiphales* (Jørgensen, 1992)*.* However, the extent to which host susceptibility factors are required for beneficial symbioses is unknown.

*MLO* encodes a plasma membrane protein with seven transmembrane helices and a C‐terminal calmodulin‐binding domain (Devoto *et al.*, 1999; Kim *et al.*, 2002). Most research on MLO1 has been on its role in powdery mildew susceptibility. Barley lines with mutations in *MLO1* have been successfully deployed to provide broad‐spectrum, durable resistance to powdery mildew since 1942 (Freisleben & Lein, 1942). Despite considerable progress on the characterization of the powdery mildew resistance phenotype and its underlying molecular mechanism, MLO1's biological role remains unclear (Kim *et al.*, 2002; Piffanelli *et al.*, 2002; Consonni *et al.*, 2010). Notably, orthologues of MLO1 are only present in plant species that can host arbuscular mycorrhizal fungi (Bravo *et al.*, 2016). The study of other MLO family members has revealed their involvement in diverse plant processes, including pollen tube reception (AtMLO7/NORTIA) and root thigmomorphogenesis (Kessler *et al.*, 2010; Bidzinski *et al.*, 2014). The ancestral function of MLO proteins is unknown, but their presence in green algae suggests that the so‐far characterized roles are derivative (Jiao *et al.*, 2011). In *Arabidopsis thaliana*, loss of MLO family members belonging to a separate clade from barley MLO1 confers resistance to powdery mildew in a redundant fashion (Kusch *et al.*, 2016; Kuhn *et al.*, 2017). Interestingly, one of these can complement the *Atmlo7* pollen tube reception phenotype, suggesting a conserved biochemical function of MLO proteins (Jones *et al.*, 2017).

Most land plants can form an endosymbiosis with members of the *Glomeromycotina*, reflecting the ancient origins of this interaction (Remy *et al.*, 1994; Spatafora *et al.*, 2016). This mutualistic relationship supplies water and nutrients – particularly phosphate, but also nitrogen and zinc – to the host plant (Cooper & Tinker, 1978; Govindarajulu *et al.*, 2005; Watts‐Williams & Cavagnaro, 2012). Several key components of the signalling pathway required for establishing mycorrhizal symbioses (the common SYM pathway) are conserved in green algae, suggesting that land plant ancestors were preadapted for symbiosis (Delaux *et al.*, 2013 2015). Furthermore, MLO1 was previously suggested to be required for colonization by arbuscular mycorrhizal fungi (Ruiz‐Lozano *et al.*, 1999). By contrast, a recent study found that a *Hvmlo1* mutant had increased mycorrhizal colonization (Hilbert *et al.*, 2019). These studies were limited to a single allele evaluated at a single time point. Another study of an MLO from a different clade found no evidence for a role in arbuscular mycorrhization of pea (Humphry *et al.*, 2011).

Using time‐course analyses of mutants from barley, wheat, and *Medicago truncatula* and transcript profiling of *Hvmlo1* mutant mycorrhizal roots, we reveal a clear role for MLO in the early stages of arbuscular mycorrhizal colonization in angiosperms. The findings suggest a role for clade IV MLOs in the establishment of beneficial fungal symbiosis, which has been exploited by pathogens such as powdery mildew.

## Materials and Methods

### Phylogenetic analyses

Sequences were aligned using Muscle (Edgar, 2004), and a maximum likelihood tree was constructed using Mega X following a Jones–Taylor–Thornton model with 100 bootstrap replications (Kumar *et al.*, 2018). Protein sequences and references are in Supporting Information Methods S1.

### Barley, wheat and *M*. *truncatula* mutant molecular analyses

Details of the barley (*Hordeum vulgare* L.) and wheat (*Triticum aestivum* L.) *mlo* mutants used are in Methods S2. All primers are listed in Methods S3. To identify homozygous *M. truncatula* Gaertn. *mlo8 Tnt1‐*insertion mutants, DNA was extracted (DNeasy 96 Plant Kit; Qiagen), and genotyping of segregating seedling populations was performed by PCR. To detect *MtMLO8* cDNA, RNA was extracted from mycorrhizal *M. truncatula* roots (Plant RNeasy Kit; Qiagen). DNases were removed (Turbo DNA‐free; Ambion), and 150 ng of RNA was retrotranscribed (SuperScript IV reverse transcriptase; Invitrogen). cDNA was amplified by RT‐PCR (Phusion High Fidelity Polymerase kit; New England Biolabs, Ipswich, MA, USA).

### Plant growth conditions and mycorrhization and powdery mildew assays

For mycorrhization experiments, barley, wheat, and *M. truncatula* were grown in pots containing 80–90% Terragreen/sand and 10–20% mycorrhizal inoculum (homogenized soil substrate containing *Allium schoenoprasum* roots colonized by *Rhizophagus irregularis* DAOM 197198). Full descriptions of seed sterilization, soils substrates, and growth conditions for mycorrhization and powdery mildew assays are in Methods S4.

### *Agrobacterium* *rhizogenes*‐mediated gene transfer

*Agrobacterium rhizogenes*‐mediated gene transfer (Boisson‐Dernier *et al.*, 2001), was performed using strain AR1193. The promoter‐GUS construct *pMtMLO8:GUS* was generated using the Gateway^®^ system (Primrose & Twyman, 2013): entry vector pDONR207; destination vector 243_pKGW‐GGRR. The 1983 bp promoter region of *MtMLO8* (Medtr3g115940) was amplified using primers with Gateway‐compatible end sequences (Methods S3). The complementation construct *pMtMLO8:MtMLO8* was generated using the Golden Gate system (Engler *et al.*, 2009) using the same *MtMLO8* promoter region fused to the 1656 bp coding region. Domesticated DNA parts were synthesized by GeneArt^®^ (Life Technologies, Carlsbad, CA, USA). pL2V‐50507 was used for the L2 backbone, with *pLjUBIQUTIN:DsRed:t53S* placed in position 1, and *pMtMLO8:MLO8:t35S* in position 2.

### Fungal staining and quantification

Staining (ink, GUS, and WGA‐Alexa488) and visualization methods are summarized in Methods S5. Samples were blinded, and mycorrhizal colonization structures quantified using the gridline intersect method (Giovannetti & Mosse, 1980). Powdery mildew fungal structures were quantified by calculating the percentage host cell entry (the ratio of germinated spores : colony formation).

### Gene expression analyses

RNAs were extracted from root tissue (Plant RNeasy Kit; Qiagen) and were treated with DNase (RNase‐Free DNase Set; Qiagen). Methods for RT‐qPCR and transcriptome analyses using RNA‐sequencing can be found in Methods S6.

## Results

**Figure 1:**
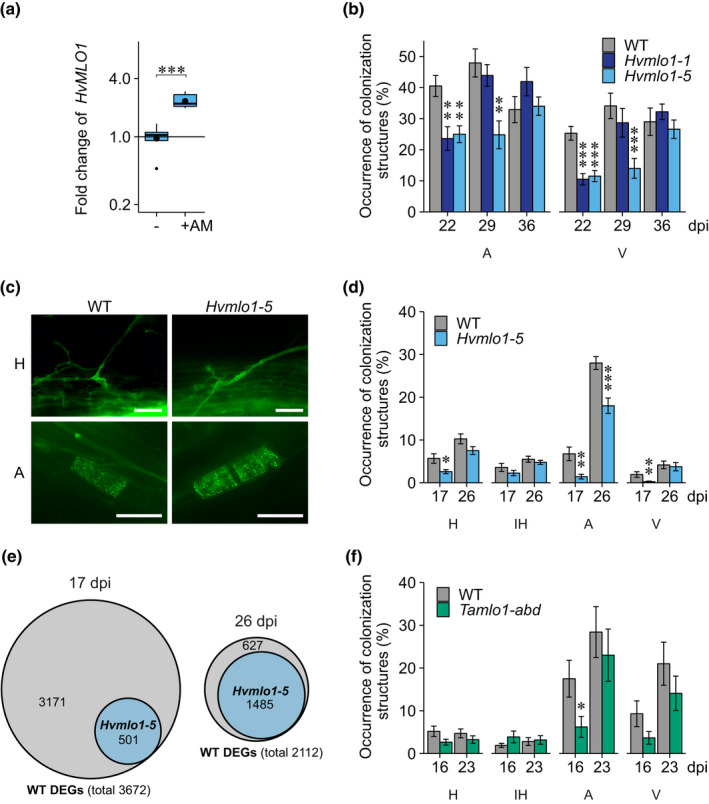
Mycorrhizal colonization in barley (*Hordeum vulgare*) and wheat (*Triticum aestivum*) *mlo1* mutants. (a) Relative expression of *HvMLO1* in wild‐type (WT) barley cv Ingrid roots without (−) and with *Rhizophagus irregularis* (+AM) at 22 d post inoculation (dpi). Relative expression levels were measured by RT‐qPCR and normalized to *HvEF1alpha* (barley). Statistical comparisons were made relative to nonmycorrhizal root samples. Boxplots represent means (large black dots), medians (black lines), 25–75% quartile (box), and upper/lower quartile ±1.5 × interquartile range (whiskers) of eight biological replicates (Student's *t*‐test: ***, *P* < 0.001). (b, d, f) Quantification of arbuscular mycorrhizal colonization structures in barley cv Ingrid and wheat cv KN1999 WT and *mlo1* roots after inoculation with *R. irregularis*: hyphopodia (H), intraradical hyphae (IH), arbuscules (A), and vesicles (V). The binomial occurrences of mycorrhizal structures are shown as a percentage of the total number of root sections assessed. Statistical comparisons have been made to the WT. Values are the mean of 12 biological replicates ± 1 SEM (error bars) (General Linear Model with a logit link function; ANOVA; *, *P* < 0.05; **, *P* < 0.01; ***, *P* < 0.001). (c) Appearance of hyphopodia (H) and arbuscules (A) in WT and *Hvmlo1‐5* roots at 17 dpi with *R. irregularis*. Mycorrhizal fungal structures were stained using Alexa Fluor 488 wheat germ agglutinin. Photographs indicate representative examples from 40 observations. Bars, 50 μm. (e) Venn diagrams showing the number of differentially regulated genes (DEGs) in mycorrhizal roots relative to uninoculated roots in WT and the proportion of genes similarly responding in *Hvmlo1‐5* using a cut‐off of fold change > 2; FDR‐corrected *P‐*value < 0.05.

To assess a potential function for MLO1 in mycorrhization, we compared expression levels of barley *MLO1* (*HvMLO1*) in mycorrhizal and nonmycorrhizal roots (Fig. 1a)*.* At 22 d post‐inoculation (dpi) with *R. irregularis*, RT‐qPCR analysis indicated that *HvMLO1* was induced in mycorrhizal roots. We then evaluated mycorrhizal phenotypes of barley *Hvmlo1* mutants by quantification of mycorrhizal fungal colonization structures over a time‐course. Arbuscules and vesicles were quantified at 22, 29, and 36 dpi with *R. irregularis* (20% inoculum) in barley cv Ingrid wild‐type (WT), *Hvmlo1‐1,* and *Hvmlo1‐5* roots (Fig. 1b; Gene structures in Methods S2). We found a reduction in arbuscule and vesicle occurrence in *Hvmlo1‐1* and *Hvmlo1‐5* compared to WT at 22 dpi. This difference was maintained in *Hvmlo1‐5* at 29 dpi, whereas *Hvmlo1‐1* showed a similar phenotype to WT. By 36 dpi, no significant difference in mycorrhization between the lines was detectable. WT roots were fully colonized by mycorrhizal fungi by 22 and 29 dpi, whereas the *Hvmlo1* mutants established full colonization between 29 and 36 dpi. Overall, reduced mycorrhization was more evident at early time points, suggesting that mycorrhizal development in *Hvmlo1* is delayed but later recovers to WT levels.

To further assess the mycorrhizal phenotype of *Hvmlo1* during the early stages of colonization, we performed a detailed analysis of symbiotic structures (hyphopodia, intraradical hyphae, arbuscules and vesicles) at early time points using less *R. irregularis* inoculum (10% inoculum) (Fig. 1c,d). At 17 dpi, *Hvmlo1‐5* showed a significant reduction in the occurrence of hyphopodia, arbuscules, and vesicles compared to WT. Similar to the previous experiment, by 26 dpi, *Hvmlo1‐5* showed a significant reduction in only arbuscule occurrence compared to WT. A similar phenotype was observed between WT and *Hvmlo1‐5* in a barley cv Pallas near‐isogenic line, in two separate experiments (Fig. S1a), suggesting the phenotype is stable across different genetic backgrounds. We observed no differences in the morphology of hyphopodia or arbuscules in *Hvmlo1‐5* mutants (Fig. 1c).

To gain insight into the nature of the delayed colonization of *Hvmlo1‐5*, we carried out comparative transcriptomic analyses at 17 dpi and 26 dpi with *R. irregularis* (Figs 1e, S2)*.* At 17 dpi, there were considerably fewer differentially regulated genes (DEGs) in *Hvmlo1‐5* than WT, with 86% (3171/3672) of all WT DEGs not responding in *Hvmlo1‐5.* By 26 dpi, despite the recovery of observable colonization in the mutant, there was still a substantial (*c.* 30%) reduction of the number of DEGs in *Hvmlo1‐5* relative to WT, suggesting persistent perturbation of the mycorrhizal interaction.

To further investigate the nature of the differences in *Hvmlo1‐5,* we examined the expression of potential barley orthologues of genes known to be involved in mycorrhizal symbiosis in other species*.* Amongst the genes induced more than two‐fold in WT – but not in *Hvmlo1‐5* – at 17 dpi, were genes required for early colonization and components of the common signalling pathway required for nodulation and mycorrhization, *VAPYRIN, NSP1, NSP2, DMI2, IPD3* and *NOPE1* homologues (Catoira *et al.*, 2000; Ané *et al.*, 2002; Lévy *et al.*, 2004; Messinese *et al.*, 2007; Pumplin *et al.*, 2010; Murray *et al.*, 2011; Nadal *et al.*, 2017) (Tables 1, S1, S2; Fig. S3). This suggests that *MLO1* is required for timely or full‐activation of early processes of mycorrhizal colonization. Also, the expression of potential orthologues of several genes involved in arbuscule development and function were affected in *Hvmlo1‐5,* including *RAM1, RAD1*, *RAM2*, *EXO70I*, *STR, STR2 AMT2‐3*, *AMT3‐4* and *PT4* homologues (Table 1; Fig. S3) (Harrison *et al.*, 2002; Zhang *et al.*, 2010; Gobbato *et al.*, 2012; Wang *et al.*, 2012; Breuillin‐Sessoms *et al.*, 2015; Xue *et al.*, 2015; Zhang *et al.*, 2015). Generally, these genes were induced to a lesser extent than WT at 17 dpi, and partly or fully recovered by 26 dpi, which mirrors the initial delay and later recovery of the colonization phenotype. Together, these phenotypic and transcriptomic results indicate a role for MLO1 in the early stage of mycorrhization.

**Table 1:** Counts per million (CPM) values of potential barley (*Hordeum vulgare (Hv)*) orthologues of *Medicago truncatula (Mt) *genes previously described to be involved in mycorrhizal symbiosis in wild‐type (WT) and *Hvmlo1‐5* nonmycorrhizal and mycorrhizal roots: significant fold changes between WT and *Hvmlo1‐5* at corresponding time points and treatments are indicated.

*Mt* gene	*Mt* identifier	*Hv* identifier	Mean normalized CPMs							
			17 dpi				26 dpi			
			Nonmycorrhizal		Mycorrhizal		Nonmycorrhizal		Mycorrhizal	
			WT	*Hvmlo1‐5*	WT	*Hvmlo1‐5*	WT	*Hvmlo1‐5*	WT	*Hvmlo1‐5*
*VAPYRIN*	Medtr6g027840	HORVU4Hr1G002180	14.3	11.0	32.2	16.2 ***	17.0	18.8	62.4	63.6
*NSP1*	Medtr8g020840	HORVU2Hr1G104160	1.0	2.6	4.0	2.2 *	6.0	6.9	8.9	8.6
*NSP2*	Medtr3g072710	HORVU4Hr1G061310	2.7	3.2	11.2	2.5 ***	4.5	5.8	47.2	57.3
*DMI1*	Medtr2g005870	HORVU5Hr1G120340	28.3	33.3	44.2	33.2 **	61.1	62.1	80.6	74.6
*DMI2*	Medtr5g030920	HORVU2Hr1G058820	11.1	17.6	72.8	29.2 ***	57.0	71.3	181.0	195.0
*DMI3*	Medtr8g043970	HORVU1Hr1G068660	14.0	17.0	23.8	14.9 **	38.4	35.2	52.3	48.6
*IPD3*	Medtr5g026850	HORVU7Hr1G008420	12.6	19.8	56.4	22.9 ***	54.7	56.7	158.8	138.8
*NOPE1*	Medtr3g093270 Medtr3g093290	HORVU2Hr1G005470	9.1	10.1	53.5	18.5 ***	18.8	15.9	121.8	149.8
*RAD1*	Medtr4g104020	HORVU3Hr1G088780	0.3	0.5	7.6	0.4 ***	1.5	1.6	16.9	15.6
*RAM2*	Medtr1g040500	HORVU4Hr1G011110	5.3	4.7	30.6	5.5 ***	7.7	8.5	77.7	68.1
*EXO70I*	Medtr1g017910	HORVU7Hr1G052100	0.1	0.1	11.6	2.2 ***	0.1	0.1	34.0	37.4
*STR*	Medtr8g107450	HORVU5Hr1G060690	1.3	0.5	14.5	1.7 ***	0.7	0.4	56.1	50.4
*STR2*	Medtr5g030910	HORVU3Hr1G066220	0.0	0.0	26.2	2.5 ***	0.1	0.2	83.3	84.2
*AMT2‐3*	Medtr8g074750	HORVU3Hr1G082610	0.0	0.0	14.3	0.7 ***	0.0	0.0	53.8	45.5
*AMT2‐4*	Medtr7g115050	HORVU5Hr1G095030	0.0	0.0	3.0	0.5 ***	0.0	0.0	9.8	12.0
*PT4*	Medtr8g074750	HORVU6Hr1G058690	0.0	0.0	3.9	1.0	0.0	0.0	16.6	21.0

FDR‐corrected *P‐*value; *, *P* < 0.05; **,* P* < 0.01; ***, *P* < 0.001.

dpi, days post inoculation.

**Figure 2:**
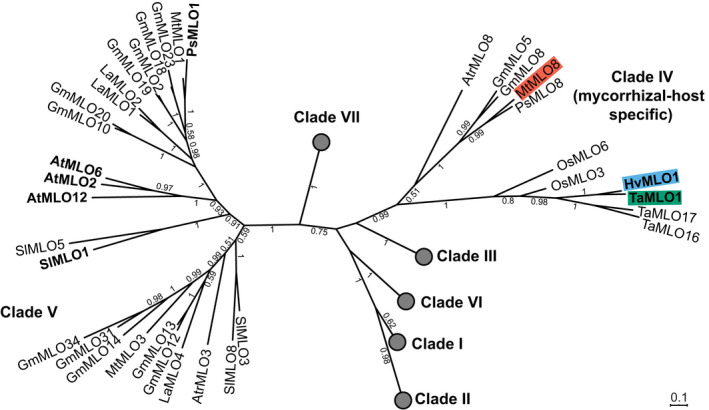
MLO phylogeny. A phylogenetic tree showing the relationship between MLO proteins from *Amborella trichopoda* (Atr), *Arabidopsis thaliana* (At),* Beta vulgaris* (Bv),* Dianthus caryophyllus* (Dc),* Glycine max* (Gm),* Hordeum vulgare* (Hv),* Lupinus angustifolius* (La),* Medicago truncatula* (Mt),* Oryza sativa* (Os),* Pisum sativum* (Ps),* Physcomitrella patens* (Pp),* Solanum lycopersicum* (Sl) *and Triticum aestivum* (Ta)*.* The *T. aestivum* MLO protein dataset often included near‐identical homoeoalleles (A, B and D copies); therefore, for simplicity, only one homoeoallele was included in the phylogenetic analysis (A homoeoallele where possible). The tree was generated using Mega X from an amino acid alignment. The phylogenetic tree was calculated via the maximum likelihood method, using Jones–Taylor–Thornton modelling with 100 bootstrap replications. Bootstrap values > 0.5 are shown. The scale bar indicates the evolutionary distance based on the amino acid substitution rate. Circles indicate collapsed clades; known powdery mildew susceptibility factors are labelled in bold, and candidate MLO proteins for a role in mycorrhization are highlighted.

To investigate the conservation of function for this MLO, we used phylogenetic analysis to identify potential functional orthologues of HvMLO1 (Fig. 2). The analysis included mycorrhizal host species: basal angiosperm *Amborella trichopoda*; dicots *Glycine max*, *M. truncatula*, *Pisum sativum,* and *Solanum lycopersicum*; and monocots *H. vulgare*, *Oryza sativa,* and *T. aestivum*, as well as nonmycorrhizal host species: moss *Physcomitrella patens*; and dicots *Arabidopsis thaliana*, *Beta vulgaris*, *Dianthus caryophyllus,* and *Lupinus angustifolius*. As previously shown (Kusch *et al.*, 2016), the phylogenetic analysis supported the view that embryophyte MLO proteins diverged into seven clades. HvMLO1 groups in clade IV, which is comprised only of species that are mycorrhizal‐hosts, while powdery mildew susceptibility factors identified in monocots and dicots are found in clades IV and V, respectively.

The wheat functional orthologue of HvMLO1, TaMLO1, is a powdery mildew host susceptibility factor (Wang *et al.*, 2014). *Tamlo1‐abd* contains TALEN‐induced mutations in all three homoeoalleles (A, B, and D) (Wang *et al.*, 2014). To assess the mycorrhizal phenotype of *Tamlo1‐abd* during the early stages of mycorrhization, we performed a detailed analysis of mycorrhizal structures (hyphopodia, intraradical hyphae, arbuscules, and vesicles) at early time points similar to those used for barley (Figs 1f, S1b). At 16 dpi with *R. irregularis*, *Tamlo1‐abd* showed a significant reduction in arbuscule occurrence compared to WT. However, consistent with our findings in barley, by 23 dpi *Tamlo1‐abd* colonization levels were similar to WT. Wheat *mlo1* mutants exhibited the delayed mycorrhizal phenotype in two separate experiments, suggesting a conserved role for MLO1 in mycorrhization in cereals.

**Figure 3:**
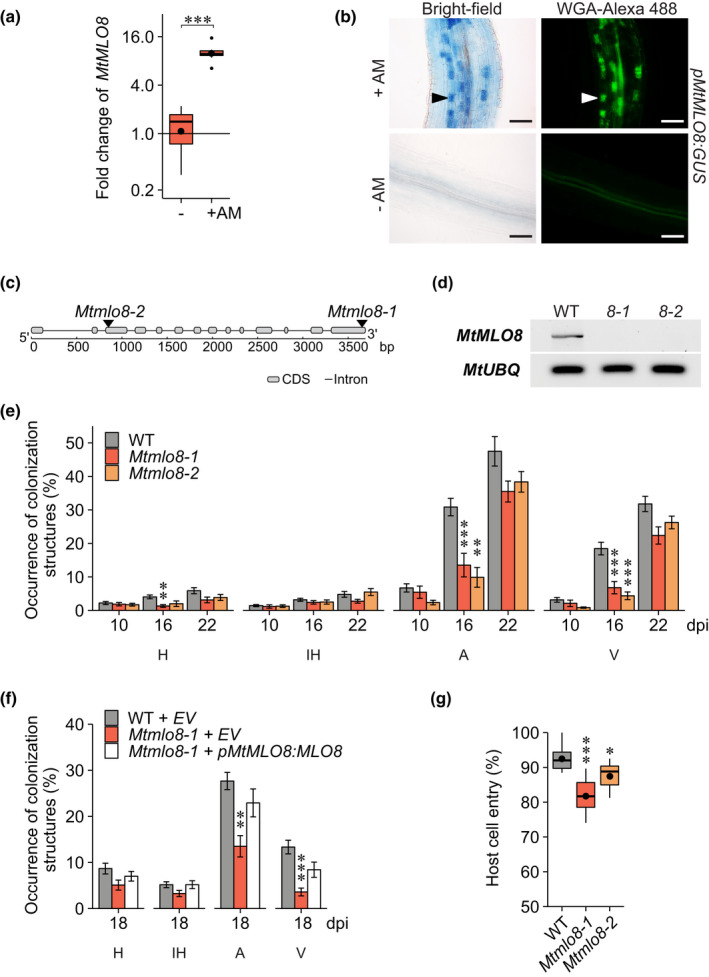
MtMLO8 is involved in mycorrhizal colonization of *Medicago truncatula.* (a) Relative expression of *MtMLO8* in wild‐type (WT) *M. truncatula* cv R108 without (−) and with *Rhizophagus irregularis* (+AM) at 22 d post inoculation (dpi). Relative expression levels were measured by RT‐qPCR and normalized to the geometric mean of *MtUBIQUITIN* and *MtPTB*. Statistical comparisons were made relative to nonmycorrhizal root samples. Boxplots represent means (large black dots), medians (black lines), 25–75% quartile (box), and upper/lower quartile ±1.5 × interquartile range (whiskers) of eight biological replicates (Student's *t*‐test: ***, *P* < 0.001). (b) Activity of the *MtMLO8* promoter in mycorrhizal (+AM) and nonmycorrhizal (−AM) WT roots at 21 dpi with *R. irregularis*, assessed using a promoter‐GUS fusion. Bright‐field and corresponding green fluorescence images of *M. truncatula* hairy roots expressing the β*‐glucuronidase* (*GUS*) gene under the control of the *MtMLO8* promoter. Mycorrhizal fungal structures were visualized using Alexa Fluor 488 wheat germ agglutinin. Arrowheads indicate examples of cells containing arbuscules. Bars, 100 μm. (c) Gene structure of *M. truncatula MtMLO8*. Arrows indicate the *Tobacco retrotransposon 1* (*Tnt1*) insertion sites in the *Mtmlo8* mutants. (d) RT‐PCR was used to detect the accumulation of the *MtMLO8* transcript in WT, *Mtmlo8‐1*, and *Mtmlo8‐2* mutant roots. *MtUBIQUITIN* was used as a constitutive control. (e) Quantification of mycorrhizal structures in *M. truncatula* cv R108 WT, *Mtmlo8‐1* and *Mtmlo8‐2* roots. (f) Complementation of the *Mtmlo8‐1* mutants and quantification of mycorrhizal structures in hairy roots. *Mtmlo8‐1* was transformed with *pMtMLO8:MtMLO8* or empty vector (EV) control, and WT were transformed with EV control. The binomial occurrences of mycorrhizal structures, hyphopodia (H), intraradical hyphae (IH), arbuscules (A), and vesicles (V), are shown as a percentage of the total number of root systems assessed. (g) Quantification of powdery mildew infection on WT, *Mtmlo8‐1* and *Mtmlo8‐2* leaves at 72 h post inoculation with *Erysiphe pisi*. The binomial occurrences of successful host cell entry (ratio of germinated spores: colony formation) are shown as a percentage of the total number of spores observed. For (e–g) statistical comparisons have been made to the WT. Bars represent the mean of 15 (e) and 10 (f) biological replicates ± 1 SEM (error bars) and boxplots (g) represent means (black dots), medians (black lines), 25–75% quartile (box), and upper/lower quartile ±1.5 × interquartile range (whiskers) of eight biological replicates (General Linear Model with a logit link function; ANOVA, *post hoc* pairwise; *, *P* < 0.05; **, *P* < 0.01; ***, *P* < 0.001).

To determine whether MLO's role in mycorrhization extends to dicots, we investigated *M. truncatula* MtMLO8, the apparent orthologue of HvMLO1, which was previously found to be conserved exclusively in mycorrhizal host plants (Bravo *et al.*, 2016). We first tested its expression in mycorrhizal roots, and we found that, like *HvMLO1, MtMLO8* was induced relative to nonmycorrhizal roots (Figs 3a, S4a), consistent with data from several studies available in the public database (Benedito *et al.*, 2008; Breakspear *et al.*, 2014; Luginbuehl *et al.*, 2017). To establish in which cell types *MtMLO8* is expressed, we studied its expression in *M. truncatula* roots using the *MtMLO8* promoter to drive *GUS* expression using the *Agrobacterium rhizogenes* mediated hairy root transformation system*. MtMLO8* promoter activity was strongly associated with cortical cells containing arbuscules (Figs 3b*,* S4b).

To investigate whether MtMLO8 functions during mycorrhization, seeds from two independent *M. truncatula* cv R108 lines carrying *Tnt1* insertions in the coding sequence of these genes were obtained, and homozygous mutants were identified (Fig. 3c,d) (Tadege *et al.*, 2008). We performed a detailed analysis of mycorrhizal structures (hyphopodia, intraradical hyphae, arbuscules, and vesicles) at multiple time points in WT, *Mtmlo8‐1,* and *Mtmlo8‐2*. Compared to WT, there was a significant reduction in arbuscules and vesicles at 16 dpi in both mutant alleles of *Mtmlo8* (Fig. 3e), consistent with our results using the orthologous mutants in barley and wheat. By 22 dpi – and at later time points (Fig. S5a) – there was no difference between the lines. We observed no impairment in the morphology of mycorrhizal structures – hyphopodia and arbuscules – in *Mtmlo8* mutants (Fig. S5b). To validate that mycorrhizal phenotype observed was due to mutations in *MtMLO8,* and not a consequence of additional *Tnt1* insertions or other mutations in the mutant backgrounds, we expressed *MtMLO8* from its native promoter in *Mtmlo8‐1* mutant roots by transformation with *Agrobacterium rhizogenes.* When arbuscule occurrence in roots of WT plants transformed with the empty vector reached *c.* 30% – corresponding to when the *Mtmlo8* phenotype could be observed – mycorrhizal structures (hyphopodia, intraradical hyphae, arbuscules, and vesicles) were quantified in the transgenic roots. The expression of *MtMLO8* under the control of its native promoter successfully complemented the *Mtmlo8‐1* mutant phenotype (Fig. 3f). In summary, these results suggest that mutations in *MtMLO8* affect the early stages of mycorrhization and delay arbuscule development.

To assess whether, like HvMLO1 and TaMLO1, MtMLO8 might also have a role in powdery mildew susceptibility, we inoculated WT and *Mtmlo8* mutant leaves with an isolate of powdery mildew that can infect *M. truncatula*, *Erysiphe pisi.* We observed a small but statistically significant reduction in the percentage of successful host cell entry in *Mtmlo8* compared to WT (Fig. 3g). This result indicates that *MtMLO8* is required not only for early mycorrhizal colonization but also for powdery mildew colonization.

## Discussion

Since MLO's function in facilitating powdery mildew infection is disadvantageous to the host, it follows that it must also fulfil some other beneficial role that explains its conservation throughout evolutionary history. The phylogenetic analysis, mutant phenotypes, and gene expression studies presented here point to a symbiotic role for members of the MLO clade IV and suggest that – at least in part – selection to maintain this gene is related to its function in mycorrhizal interactions. Recent evidence also suggests a positive role for MLO1 in supporting endophytic interactions with *Serendipita indica* in barley (Hilbert *et al.*, 2019). A common thread between the established roles of MLO in fungal interactions, pollen‐tube reception, and thigmotropism is touch‐sensing. Indeed, MLO proteins have been reported to accumulate at the site of powdery mildew penetration (Bhat *et al.*, 2005), as well as at the contact point between the pollen tube and synergid cell (Kessler *et al.*, 2010). These responses could result from a generic response to mechanical stimuli. Notably, a role for mechanosensing has been proposed for the receptor kinase Feronia (Hamant & Haswell, 2017), which was shown to be required for proper re‐localization of MLO7/NORTIA to the site of pollen tube contact in the synergid cell (Kessler *et al.*, 2010; Jones *et al.*, 2017). However, other cues may activate MLO, for example, the biotrophic fungi *Blumeria graminis*, *R. irregularis*, and *Serendipita indica* produce effectors that could target MLO (Kloppholz *et al.*, 2011; Akum *et al.*, 2015; Requena *et al.*, 2018; Pennington *et al.*, 2019; Saur *et al.*, 2019). Regardless of how it is activated, it will be of interest to study the localization of MLO1 or its orthologues during hyphopodium formation.

The mycorrhizal phenotype of the *mlo* mutants examined was not severe, but may be expected to have measurable consequences on fitness in a natural setting with competition for limited resources. Furthermore, global gene expression was still affected at the later time point, so a detailed study of growth and nutrient uptake characteristics of the mutant may show differences. Also, the increased expression of other MLOs during mycorrhization in *M. truncatula* (Fig. S3a) suggests that – as for other *mlo* phenotypes such as powdery mildew resistance (Kuhn *et al.*, 2017) and synergid cell reception (Jones & Kessler, 2017) – MLOs act redundantly, with different MLOs potentially contributing unequally across different cell types and developmental stages. It would be interesting to test whether *MtMLO3* – which was highly induced during mycorrhization – and *MLO* genes with high epidermal expression (Breakspear *et al.*, 2014), function together with *MtMLO8* during arbuscular mycorrhizal colonization. The powdery mildew phenotype of *Mtmlo8* was relatively minor. Additional MLO genes may contribute to powdery mildew susceptibility – as is the case in *Arabidopsis thaliana* – for example, *MtMLO1*, the apparent orthologue of *PsMLO1,* which is involved in powdery mildew susceptibility in pea (Humphry *et al.*, 2011; Pavan *et al.*, 2011). Notably, the narrow time window for mycorrhizal phenotype definition and potential functional redundancy with gene family members could explain why MLO has not so far been identified in mutagenesis screens.

Despite having yield penalties and potentially enhancing susceptibility to necrotrophic pathogens, mutants for MLO have been deployed in agriculture for decades (Brown, 2002; McGrann *et al.*, 2014). Understanding the role of MLO in symbiotic interactions with mycorrhizal fungi is therefore vital information for sustainable agriculture (Martin *et al.*, 2018). Our detailed phenotypic and transcriptomic analyses in both cereals and *M. truncatula* provide the basis for further elucidation of MLO function in mycorrhization and how plants balance this beneficial association with susceptibility to parasitic powdery mildews. Since mycorrhizal fungi and powdery mildew respectively infect root and shoot, it may be possible to generate genotypes that could fully support mycorrhiza while remaining nonhosts for powdery mildew.

## Author contributions

CNJ performed experimental work and data analyses. MC, JDM and CJR supervised and co‐directed the research. CNJ, JDM, CJR and MC wrote the manuscript.

## Supporting information

Please note: Wiley Blackwell are not responsible for the content or functionality of any Supporting Information supplied by the authors. Any queries (other than missing material) should be directed to the *New Phytologist* Central Office.

**Fig. S1** Mycorrhization in barley (*Hordeum vulgare*) cv Pallas and wheat (*Triticum aestivum*) cv KN199 wild‐type (WT) and *mlo *mutants.
**Fig. S2** Proportion of differentially expressed genes (DEGs) in barley (*Hordeum vulgare*) cv Ingrid wild‐type (WT) and *Hvmlo1‐5 *mutants in mycorrhizal roots relative to uninoculated roots.
**Fig. S3** Relative expression of potential barley (*Hordeum vulgare*) orthologues of genes known to be involved in mycorrhizal symbiosis.
**Fig. S4** *Medicago truncatula* MLO gene expression.
**Fig. S5** Mycorrhization in *Medicago truncatula* wild‐type (WT) and *Mtmlo8* mutants.

**Methods S1** MLO protein sequences.
**Methods S2** Gene structure of barley (*Hordeum vulgare*) and wheat (*Triticum aestivum*) *MLO1.*
**Methods S3** Primer sequences.
**Methods S4** Sterilization methods and growth conditions.
**Methods S5** Staining and visualization methods.
**Methods S6** Gene expression analyses.

**Table S1** Normalized read counts for RNA‐seq samples obtained from barley (*Hordeum vulgare*) cv Ingrid wild‐type and *Hvmlo1‐5* roots at 17 dpi and 26 dpi with and without *Rhizophagus irregularis*.

**Table S2** RNA‐seq analysis – Fold changes (log_2_) of RNA‐seq samples obtained from barley (*Hordeum vulgare*) cv Ingrid wild‐type and *Hvmlo1‐5* roots at 17 dpi and 26 dpi with and without *Rhizophagus irregularis.*
