# Supplementary material for: Mildew Locus O facilitates colonization by arbuscular mycorrhizal fungi in angiosperms
Source: New Phytol. 2020 Feb 28;227(2):343–51. doi: 10.1111/nph.16465 (PMC7317859; doi:10.1111/nph.16465)
Supplement: Supplementary file 1 — Fig. S1 Mycorrhization in barley (Hordeum vulgare) cv Pallas and wheat (Triticum aestivum) cv KN199 wild‐type (WT) and mlo mutants. Fig. S2 Proportion of differentially expressed genes (DEGs) in barley (Hordeum vulgare) cv Ingrid wild‐type (WT) and Hvmlo1‐5 mutants in mycorrhizal roots relative to uninoculated roots. Fig. S3 Relative expression of potential barley (Hordeum vulgare) orthologues of genes known to be involved in mycorrhizal symbiosis. Fig. S4 Medicago truncatula MLO gene expression. Fig. S5 Mycorrhization in Medicago truncatula wild‐type (WT) and Mtmlo8 mutants. [file NPH-227-343-s001.pdf]

New Phytologist

***New Phytologist* Supporting Information**

Article title: Mildew Locus O facilitates colonization by arbuscular mycorrhizal fungi in angiosperms
Authors: Catherine N. Jacott, Myriam Charpentier, Jeremy D. Murray, Christopher J. Ridout
Article acceptance date: 27 January 2020

The following Supporting Information is available for this article:

Fig. S1 Mycorrhization in barley (*Hordeum vulgare*) cv. Pallas and wheat (*Triticum aestivum*) cv. KN199 wild type (WT) and *mlo* mutants

Fig. S2 Proportion of differentially expressed genes (DEGs) in barley (*Hordeum vulgare*) cv. Ingrid wild type (WT) and *Hvmlo1-5* mutants in mycorrhizal roots relative to uninoculated roots

Fig. S3 Relative expression of potential barley (*Hordeum vulgare*) orthologs of genes known to be involved in mycorrhizal symbiosis

Fig. S4 *Medicago truncatula MLO* gene expression

Fig. S5 Mycorrhization in *Medicago truncatula* wild type (WT) and *Mtmlo8* mutants

Table S1 Normalized read counts for RNA-seq samples obtained from barley (*Hordeum vulgare*) cv. Ingrid wild type and *Hvmlo1-5* roots at 17 dpi and 26 dpi with and without *Rhizophagus irregularis.*

Table S2 RNA-seq analysis **-** Fold changes (log_2) of RNA-seq samples obtained from barley (*Hordeum vulgare*) cv. Ingrid wild type and *Hvmlo1-5* roots at 17 dpi and 26 dpi with and without *Rhizophagus irregularis.*

Methods S1 MLO protein sequences

New Phytologist

Methods S2 Gene structure of barley (*Hordeum vulgare*) and wheat (*Triticum aestivum*) *MLO1*

Methods S3 Primer sequences

Methods S4 Sterilization methods and growth conditions

Methods S5 Staining and visualization methods

Methods S6 Gene expression analyses

Fig. S1 Mycorrhization in barley (*Hordeum vulgare*) cv. Pallas and wheat (*Triticum aestivum*) cv. KN199 wild type (WT) and *mlo* mutants. (a) Quantification of arbuscular mycorrhizal structures, hyphopodia (H), intraradical hyphae (IH), arbuscules (A), and vesicles (V) in barley cv. Pallas WT and *Hvmlo1-5* roots at 16 and 23 days post-inoculation (dpi) with *Rhizophagus irregularis.* **(b)** Quantification of arbuscules (A), and vesicles (V) in wheat cv. KN199 WT and *Tamlo1-abd* at 18 dpi with *R. irregularis.* The binomial occurrences of mycorrhizal structures are shown as the percentage of the total number of root sections assessed. Statistical comparisons have been made to the WT. Values are the mean of >10 biological replicates ±1SEM (error bars) (General Linear Model with a logit link function; ANOVA; *, $P < 0.05$).

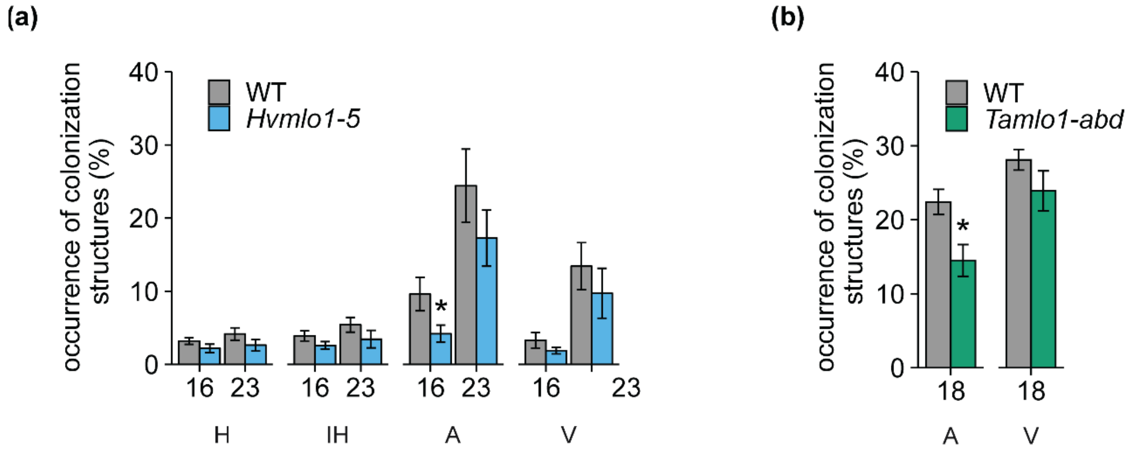

Fig. S2 Proportion of differentially expressed genes (DEGs) in barley (*Hordeum vulgare*) cv. Ingrid wild type (WT) and *Hvmlo1-5* mutants in mycorrhizal roots relative to uninoculated roots. (a) Venn diagrams and tables showing the extent of overlap of similarly responding (up- or down-regulated) DEGs between WT and *Hvmlo1-5* root at 17 and 26 dpi. (**b**) Number of up- and down-regulated genes during mycorrhization in WT and *Hvmlo1-5* roots at 17 and 26 dpi. DEGs with a fold change > 2 and FDR-corrected *P*-value < 0.05 are shown.

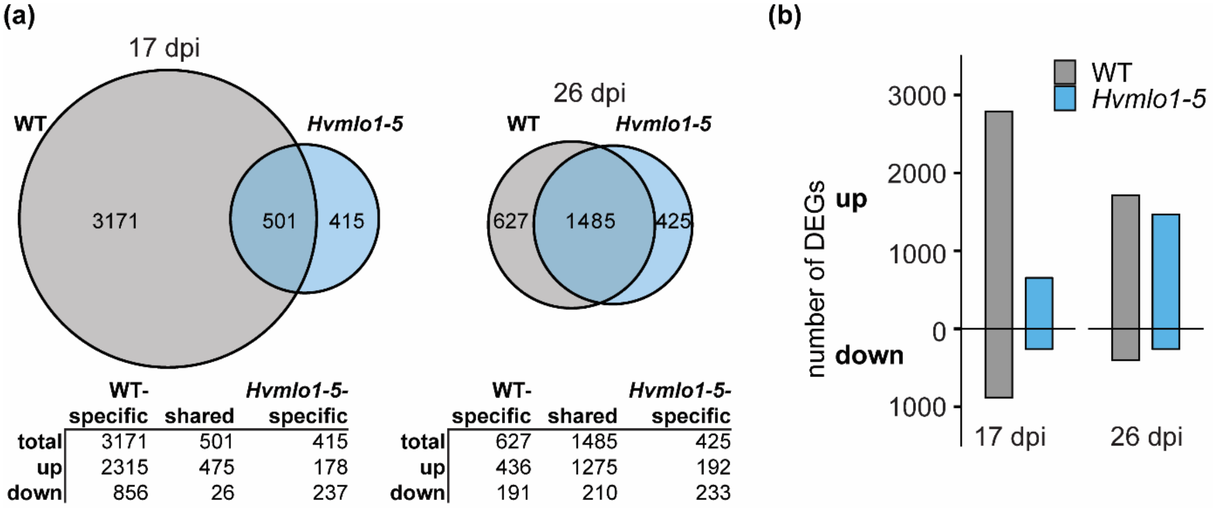

17 dpi

	WT-specific	shared	Hvmlo1-5-specific
total	3171	501	415
up	2315	475	178
down	856	26	237

26 dpi

	WT-specific	shared	Hvmlo1-5-specific
total	627	1485	425
up	436	1275	192
down	191	210	233

Fig. S3 Relative expression of potential barley (*Hordeum vulgare*) orthologs of genes known to be involved in mycorrhizal symbiosis. Relative expression of *HvVAPYRIN* **(a)**, *HvIPD3* **(b)**, *HvDMI3* **(c)**, *HvRAM1* **(d)**, *HvRAM2* **(e)** and *HvPT4* **(f)** in barley cv. Ingrid wild type (WT) and *Hvmlo1-5* roots at 17 and 26 dpi with *Rhizophgus irregularis.* Expression levels were measured by RT-qPCR and normalized to *HvEF1alpha.* Statistical comparisons were made relative to uninoculated root samples. Boxplots represent medians (black lines), 25 – 75% quartile (box), and upper/lower quartile +/- 1.5 x interquartile range (whiskers) of 4 biological replicates (Student's t-test: ***, P < 0.001).

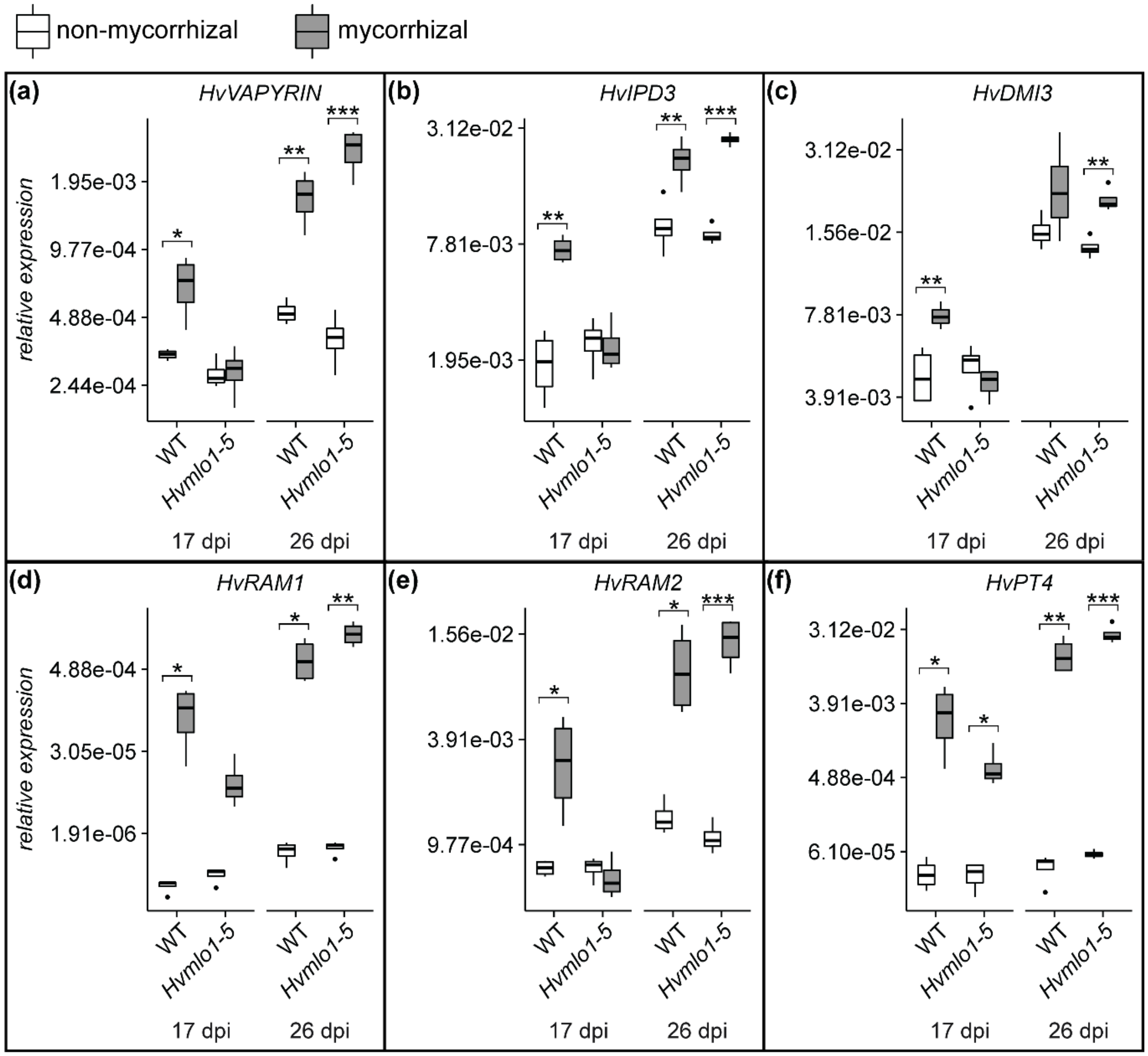

Fig. S4 *Medicago truncatula MLO* gene expression. (a) Fold change of *MtMLO* genes during mycorrhization. Gene expression was measured by RT-qPCR in *Medicago truncatula* cv. R108 wild type (WT) mycorrhizal roots relative to uninoculated roots at 22 dpi. Expression levels were measured by RT-qPCR and normalized to the geometric mean of *MtUBIQUITIN* and *MtPTB.* Statistical comparisons were made relative to uninoculated root samples. Bars represent the means of 8 biological replicates ±1SEM (error bars) (Student's *t*-test: *, $P < 0.05$; **, $P < 0.01$; ***, $P < 0.001$). **(b)** Activity of the empty vector control in mycorrhizal (+AM) and uninoculated (-AM) WT roots at 21 dpi. Bright-field and corresponding gene fluorescence images of *M. truncatula* hairy roots transformed with an empty vector construct (no promoter driving the *β-glucuronidase* (*GUS*) gene). Mycorrhizal fungal structures were visualized using Alexa Fluor 488 wheat germ agglutinin. Solid arrowheads indicate cells containing arbuscules. Scale bars = 100 µm.

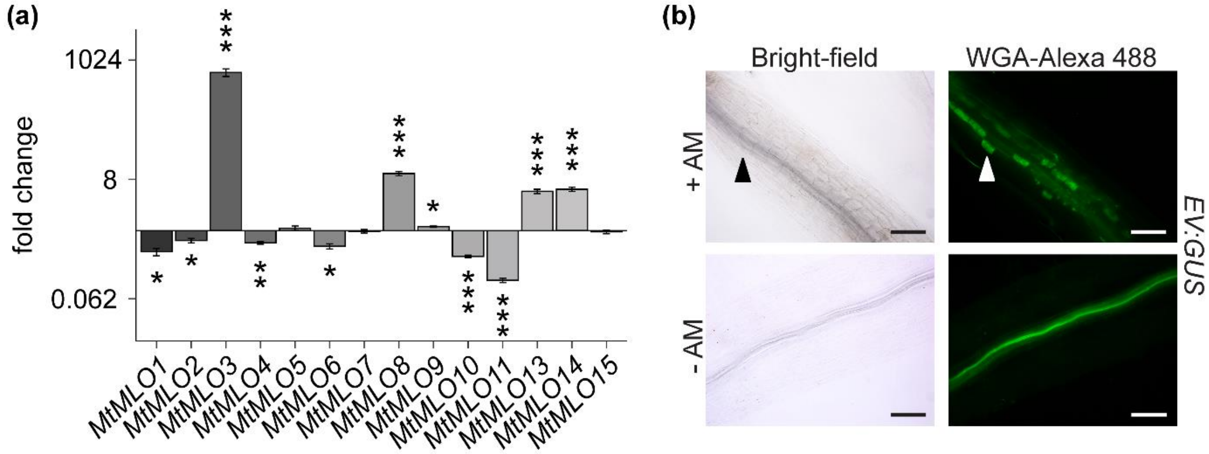

Fig. S5 Mycorrhization in *Medicago truncatula* wild type (WT) and *Mtmlo8* mutants. (a) Quantification of arbuscular mycorrhizal structures, arbuscules (A), and vesicles (V) in wild type and *Mtmlo8* roots at 28 and 38 dpi with *Rhizophagus irregularis.* The binomial occurrences of mycorrhizal structures are shown as a percentage of the total number of root sections assessed. Statistical comparisons have been made to the WT. Values are the mean of 12 biological replicates ±1SEM (error bars) (General Linear Model with a logit link function; ANOVA. **b)** Appearance of hyphopodia (H) and arbuscules (A) in WT and *Mtmlo8* mutant roots at 16 dpi with *R. irregularis.* Mycorrhizal fungal structures were visualized with ink-staining. Scale bars = 50 µm.

(a)

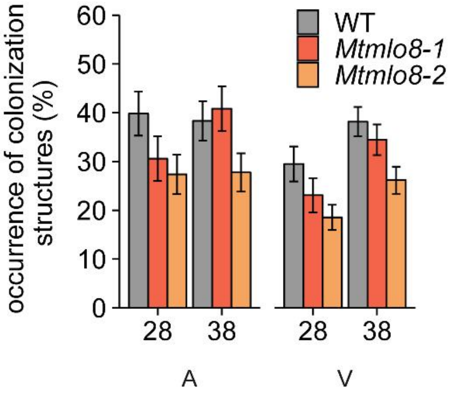

(b)

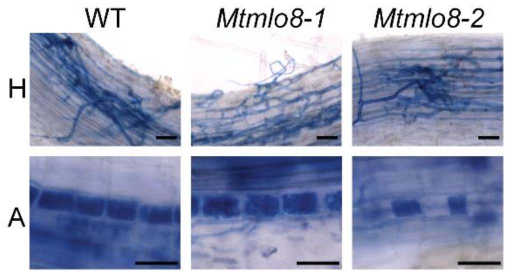

Table S1 Normalized read counts for RNA-seq samples obtained from barley (*Hordeum vulgare*) cv. Ingrid wild type and *Hvmlo1-5* roots at 17 dpi and 26 dpi with and without *Rhizophagus irregularis.* Predicted *Arabidopsis thaliana* orthologs and corresponding descriptions and primary gene symbols are shown where possible. NA indicates no predicted *A. thaliana* ortholog.

Excel file separate

Table S2 RNA-seq analysis - Fold changes (log2) of RNA-seq samples obtained from barley (*Hordeum vulgare*) cv. Ingrid wild type and *Hvmlo1-5* roots at 17 dpi and 26 dpi with and without *Rhizophagus irregularis.* At each time point (17 dpi and 26 dpi) fold changes for all genes are shown in 1. Wild type roots: nonmycorrhizal (uninoculated) versus mycorrhizal, 2. *Hvmlo1-5* roots: nonmycorrhizal (uninoculated) versus mycorrhizal, and 3. Mycorrhizal roots: wild type versus *Hvmlo1-5.* Predicted *Arabidopsis thaliana* orthologs and corresponding descriptions and primary gene symbols are shown where possible. NA indicates no predicted *A. thaliana* ortholog.

Excel file separate

Methods S1 MLO protein sequences. MLO protein sequences from *Amborella trichopoda, Arabidopsis thaliana, Beta vulgaris, Dianthus caryophyllus, Glycine max, Hordeum vulgare, Lupinus angustifolius Medicago truncatula, Oryza sativa, Pisum sativum Physcomitrella patens, Solanum lycopersicum,* and *Triticum aestivum.*

Excel file separate

Methods S2 Gene structure of barley (*Hordeum vulgare*) and wheat (*Triticum aestivum*) *MLO1*. Arrows indicate the **(a)** X-ray-induced (*Hvmlo1-1*) and Ethylmethanesulfonate-induced (*Hvmlo1-5*) mutation sites in barley cv. Ingrid and Pallas *Hvmlo1* mutants (Jørgensen, 1992) and **(b)** TALEN-induced mutation sites in the wheat cv. KN199 *Tamlo1* mutant (Wang et al., 2014).

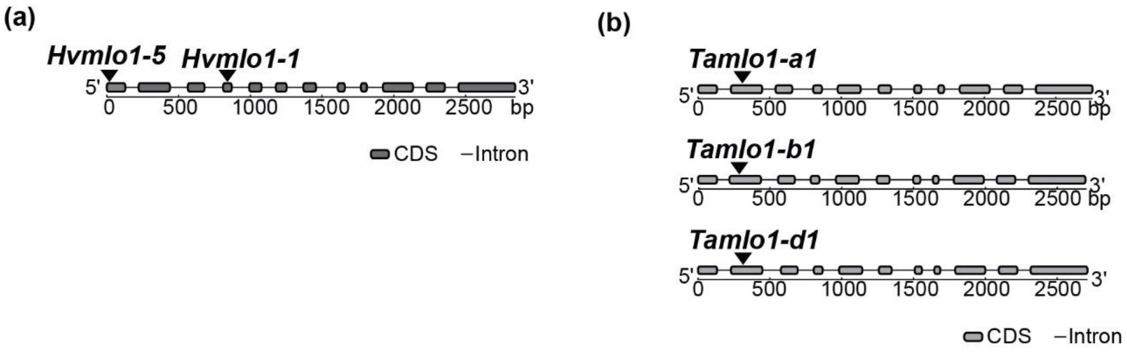

Methods S3 Primer sequences

Description	Name, reference	Sequence
**Barley**		
RT-qPCR	HvEF1a_F (Schoonbeek *et al.*, 2015)	ATGATTCCCACCAAGCCCAT
	HvEF1a_R (Schoonbeek *et al.*, 2015)	ACACCAACAGCCACAGTTTGC
	HvMLO1_F	GCAAGCCCAGCAAGTACG
	HvMLO1_R	GCGAGCACGAAGATGAAGAC
	HvVAPYRIN_F	GTGGTGGATGTCCTCCTCAAG
	HvVAPYRIN_R	CACCGACCTTCTCCAGTAACC
	HvDMI3_F	GCTAGCAGAATTCGAGCAGGT
	HvDMI3_R	CAGAGGATCTCCCTCATGTCC
	HvIPD3_F	CGCAGAGCTTCAGAGAAGACA
	HvIPD3_R	AGATGGTTGTCGAGGTCGATT
	HvRAM1_F	CCAGGCTCCCAAGATTATGAC
	HvRAM1_R	GAGTCGAAGATCGCCGAGTAG
	HvRAM2_F	GGCTGGACCCCTTCTACTTCT
	HvRAM2_R	AGGCGATGAGTCTCTGGATGT
	HvPT4_F (Zhang *et al.*, 2010)	GGATTCTTTTGCACGTTCTTGG
	HvPT4_R (Zhang *et al.*, 2010)	CCTGTCATTTGGTGTTGCAGTG

Description	Name, reference	Sequence
M. truncatula		
Genotyping *Mtmlo8* mutants	TNT1	CAGTGAACGAGCAGAACCTGTG
	NF11523_F	TCACCTTTGCACTTGCTTCA
	NF11523_R	AGAGCATGCCTTGGATGTGT
	NF15162_F	CCATGAGTGAAGCCTTGGAG
	NF15162_R	AAGGCACTTGACCCTGCATA
MtMLO8 cDNA amplification	Full_MtMLO8_F	AGCTCCTGGGGAAAGAACAT
	Full_MtMLO8_R	AAGGCTTATCAAATGAGAAGTCAATA
Gateway compatible primers (promoter-GUS)	attB_pMtMLO8_F	ggggacaagtttgtacaaaaaagcaggctTCTGGACCGGTTCAAAAATAAAA
	attB_pMtMLO8_R	GAAAGAAGAAAGCAAATTAAGAacccagctttcttgtacaaagtggtcccc
RT-qPCR	MtUBIQUITIN_F (Kakar *et al.*, 2008)	GCAGATAGACACGCTGGGA
	MtUBIQUITIN_R (Kakar *et al.*, 2008)	AACTCTTGGGCAGGCAATAA
	MtPTB_F (Kakar *et al.*, 2008)	CGCCTTGTCAGCATTGATGTC
	MtPTB_R (Kakar *et al.*, 2008)	TGAACCAGTGCCTGGAATCCT
	MtMLO1_F	TGCTTCCACAGAACAACTGC
	MtMLO1_R	CTGTGTGGTGCCATTTCTTG
	MtMLO2_F	AGACCCCTACTTGGGCTGTT
	MtMLO2_R	AATAATCCAATGGCCCATCA
	MtMLO3_F	AATTTGCATGGGAGTCTTCGT
	MtMLO3_R	TATGTTCTTCCTTGCGGTGTG
	MtMLO4_F	CTGAACGAGTGAAGCCATCAG
	MtMLO4_R	TCCATATGTGGACCAAATCCA
	MtMLO5_F	TGCAAAACTGGCGTAAAAATG
	MtMLO5_R	AGCTGTGTTATGCTCCTGCAA
	MtMLO6_F	AGCTTTTGGGATTGTGATGCT
	MtMLO6_R	GCCTTGGATGTTTGTTCATCA
	MtMLO7_F	TTGGTTTAGCGATGAGCAAGA
	MtMLO7_R	GGATGAGATGCATGATGGAAA
	MtMLO8_F	GACTATGCGGCATGGGTTTAT
	MtMLO8_R	GCAAAAGCCCATAAAGGAACA
	MtMLO9_F	TTCTGGATTGCTTTCGTTCCT
	MtMLO9_R	CTATGGCTGCATGCTTTTCAG
	MtMLO10_F	AGAAGATGGTGCCCCTTACAA
	MtMLO10_R	TGCCTTCCATCCTCGTATCTT
	MtMLO11_F	ATTCCTTTCCCGCACTTATGA
	MtMLO11_R	CAGTTTAGTCCCCGCAAGAAG
	MtMLO13_F	TTTTGGGTTGCTTTCATTCCT
	MtMLO13_R	TTTGCCTTGTATGGCTGAATG
	MtMLO14_F	TGTCAATGGTTGGCACACATA
	MtMLO14_R	TCACCTTGTATGGCTGAATGC
	MtMLO15_F	TTACACTTCGCAAAGGCTTCA
	MtMLO15_R	CCGACAACAAATCCCCATAGT

Methods S4 Sterilization methods and growth conditions

<table>
<tr><th>Figures</th><th>Species</th><th>Sterilization</th><th>Growth conditions</th></tr>
<tr><td>1a,b</td><td>Barley</td><td rowspan="3">2% sodium hypochlorite for 4 min, 5 x rinse sterile dH_2O. Seeds were spread onto plates containing sterilized filter paper, then germinated for 2 days in darkness at 23°C</td><td>Germinated seedlings were transferred to pots containing 80% sterilized Terragreen/Sand (1:1 mix of Terragreen (Oil-dri UK ltd, UK) and sharp sand) and 20% mycorrhizal inoculum (soil substrate containing Allium schoenoprasum roots colonized by Rhizophagus irregularis strain DAOM 197198 for 8 weeks). Plants were grown in a glasshouse with no additional heat or light during June – August 2018 (temperature range: 14°C - 48°C; average temperature: 28°C). Plants were watered with nutrient-free reverse osmosis (RO) water.</td></tr>
<tr><td>1c,d,e</td><td>Barley</td><td>Germinated seedlings were transferred plates containing modified Fahraeus plant agar medium (ModFP) with Augmentin (50 µg/ml), grown in a controlled-environment room at 23°C (16-h photoperiod, and 300 mmol m^{-2} s^{-1}) for 3 days, then transferred to 90% sterilized Terragreen/Sand and 10% mycorrhizal inoculum. Plants were grown in a Weiss Technik growth cabinet at 35°C/20°C (35% RH day, 50 % RH night; 16-h photoperiod, and 500 µmol m^{-2} s^{-1}, average temperature: 30°C) and harvested at 17 dpi and 26 dpi. Plants were watered with nutrient-free RO water.</td></tr>
<tr><td>1f, S1</td><td>wheat, barley</td><td>Germinated seedlings were transferred to pots containing 90% sterilized Terragreen/Sand and 10% mycorrhizal inoculum. Plants were grown in a glasshouse with no additional heat or light during June – August 2018 (temperature range: 14°C - 48°C; average temperature: 28°C). Plants were watered with nutrient-free RO water.</td></tr>
<tr><td>3a,e, S4</td><td>M. truncatula</td><td rowspan="3">Concentrated sulphuric acid for 8 min, 5 x rinse sterile dH_2O, 10% sodium hypochlorite for 4 min, 5 x rinse sterile dH_2O. Seeds were imbibed in sterile dH_2O containing Nystatin (5 µg/ml) and Augmentin (50 µg/ml) for 5 h, then placed onto water agar containing Nystatin (5 µg/ml) and Augmentin (50 µg/ml). Seeds were stratified for 5 days in darkness at 4°C, then seeds germinated overnight in darkness at 23°C.</td><td>Germinated seedlings were grown on plates containing modFP in a controlled-environment room for 7 days at 23°C (16-h photoperiod, and 300 mmol m^{-2} s^{-1}) then transferred to pots containing 90% sterilized Terragreen/Sand and 10% mycorrhizal inoculum (soil substrate containing Allium schoenoprasum roots colonized by Rhizophagus irregularis strain DAOM 197198 for 8 weeks). Plants were grown in a controlled environment room at 22°C (80% humidity, 16-h photoperiod, and 300 mmol m^{-2} s^{-1}).</td></tr>
<tr><td>3b,f</td><td>M. truncatula</td><td>Roots of germinated seedlings were transformed by Agrobacterium rhizogenes-mediated gene transfer (Boisson-Dernier et al., 2001) and grown for 3 weeks on plates containing modFP in a controlled-environment room at 23°C (16-h photoperiod, and 300 mmol m^{-2} s^{-1}). Seedlings were transferred to pots containing 90% sterilized Terragreen/Sand and 10% mycorrhizal inoculum. Plants were grown in a controlled environment room at 22°C (80% humidity, 16-h photoperiod, and 300 mmol m^{-2} s^{-1}). Plants were watered with nutrient-free RO water.</td></tr>
<tr><td>3g</td><td>M. truncatula</td><td>Germinated seedlings were transferred to soil and grown for 3 weeks in a Schneider growth cabinet at 18°C (16-h photoperiod 300 mmol m^{-2} s^{-1}). Detached M. truncatula leaves were placed on distilled water agar plates containing benzimidazole (10 µg/ml) and inoculated with by blowing fresh Erysiphe pisi (Ep) CJ001 spores into an inoculation tower.</td></tr>
</table>

Methods S5 Staining and visualization methods

Figures	Description	Staining	Visualization
1b,d,f, 3e,f, S1, S4	Ink-staining of mycorrhizal structures	Roots were washed in tap water and then incubated in 10% KOH for 5 min at 96 °C, rinsed in dH_2O, then stained using 5% black ink (Waterman, France) and 5% acetic acid for 3 min at 96 °C. Roots were de-stained in dH_2O for 1 day.	M80 microscope (Leica)
1c, 3b	Fluorescence staining of mycorrhizal structures	Roots were washed in sterile dH_2O, placed in 50% ethanol overnight at room temperature, then placed in 20% KOH for 2 days at room temperature. Root samples were rinsed in sterile dH_2O, incubated in 0.1M HCL for 3 h, rinsed in sterile dH_2O, then rinsed in 1 x phosphate-buffered saline (PBS). Root samples were stained overnight with Wheat Germ Agglutinin (WGA) labeled with Alexa Fluor 488 by incubation in a 1 x PBS solution containing 0.4 µg/ml Alexa Fluor 488 WGA in darkness at 23°C. Samples were de-stained and stored in 1 x PBS at 4°C.	DM 6000 microscope (Leica) and a DFC420 colour camera (Leica).
3b	GUS-staining	Roots were washed in sterile dH_2O then 1 x phosphate buffer pH 7.0 (NaH_2PO_4-Na_2HPO_4). Roots were vacuum infiltrated for 30 min in GUS staining solution (50 mM phosphate buffer pH 7.0, 0.5 mM $K_3Fe(CN)_6$, 0.5 mM $K_4Fe(CN)_6$, 50 mM EDTA, 3% sucrose and 2 mM X-Gluc (5-bromo-4-chloro-3-indolyl-beta-D-glucuronide), incubated in the dark at 37°C for 2 h, then rinsed in 20% ethanol for 20 mins, 50% ethanol for 20 mins, and 70% ethanol for 20 mins.	DM 6000 microscope (Leica) and a DFC420 colour camera (Leica).
3d	Trypan blue staining of powdery mildew	Leaves were placed in 70% ethanol and agitated at 150 rpm. After 6 h, leaves were placed in 100% ethanol and agitated at 150 rpm. Ethanol was regularly changed until chlorophyll was removed and the leaves were white. Leaves were placed in lactoglycerol (1:1:1 lactic acid: glycerol: dH2O) for 30 min, then stained using Trypan blue stain (0.1% Trypan blue in lactoglycerol) for 10 min.	Vickers microscope

Methods S6 Gene expression analyses. Methods for RT-qPCR and transcriptome analyses.

For RT-qPCR, 150 ng of RNA was retrotranscribed (SuperScript IV reverse transcriptase, Invitrogen). For RT-qPCR analysis, gene expression was monitored by SYBR Green-based quantitative PCR using a LightCycler LC480 system using gene-specific primers **(Methods S3)**. Data were analyzed according to the $2^{-\Delta\Delta CT}$ method (Livak & Schmittgen, 2001) using amplification efficiency corrections.

For transcriptome analysis, RNA sequencing was performed by Novogene (Cambridge, UK). mRNA libraries were prepared with the Illumina TruSeq® Stranded mRNA HT technology with a paired-end 150 bp (PE 150) strategy. To obtain pseudo-counts, Kallisto (Bray *et al.*, 2016) version 0.44 was used. Coding sequences (CDS) from the genome of barley cv. Morex (Beier *et al.*, 2017) were used as reference. For subsequent analysis, expression values from all splice forms were combined to one expression value per gene. Differential gene expression analysis was performed using DEGUST v.3.2.0 (Powell *et al.*, 2019) and edgeR was used as the normalization method. Genes with an assigned false discovery rate (FDR)-corrected P-value >0.05 were discarded from further analysis. Normalized read counts for all treatments can be found in **Table S1** and are deposited on the Gene Expression Omnibus (***GEO***) database: accession number GSE144199.

References

Beier S, Himmelbach A, Colmsee C, Zhang X-Q, Barrero RA, Zhang Q, Li L, Bayer M, et al. 2017. Construction of a map-based reference genome sequence for barley, *Hordeum vulgare L*. *Scientific data* **4**: 1-24

Bray N, Pimentel H, Melsted P and Pachter L. 2016. Near-optimal RNA-Seq quantification with kallisto. *Nat. Biotechnol* **34**: 525-527

Boisson-Dernier A, Chabaud M, Garcia F, Bécard G, Rosenberg C and Barker DG. 2001. *Agrobacterium rhizogenes*-Transformed Roots of *Medicago truncatula* for the Study of Nitrogen-Fixing and Endomycorrhizal Symbiotic Associations. *Molecular Plant-Microbe Interactions* **14**: 695-700

Jørgensen IH. 1992. Discovery, characterization and exploitation of Mlo powdery mildew resistance in barley. *Euphytica* **63**(1-2): 141-152.

Kakar K, Wandrey M, Czechowski T, Gaertner T, Scheible W-R, Stitt M, Torres-Jerez I, Xiao Y, et al. 2008. A community resource for high-throughput quantitative RT-PCR analysis of transcription factor gene expression in *Medicago truncatula*. *Plant Methods* **4**: 18-18

Livak KJ and Schmittgen TD. 2001. Analysis of Relative Gene Expression Data Using Real-Time Quantitative PCR and the $2^{-\Delta\Delta CT}$ Method. *Methods* **25**: 402-408

Powell D, Milton M, Perry A and Kim S. 2019. Degust 3.2.0 (Version 3.2.0). In, edited by.: ZenodoI

Wang Y, Cheng X, Shan Q, Zhang Y, Liu J, Gao C, Qiu J-L. 2014. Simultaneous editing of three homoeoalleles in hexaploid bread wheat confers heritable resistance to powdery mildew. *Nature Biotechnology* **32**(9): 947.
